# Maternally Inherited Diabetes Mellitus Associated with a Novel m.15897G>A Mutation in Mitochondrial tRNA^Thr^ Gene

**DOI:** 10.1155/2020/2057187

**Published:** 2020-01-30

**Authors:** Ke Li, Lijun Wu, Jianjiang Liu, Wei Lin, Qiang Qi, Tianlan Zhao

**Affiliations:** ^1^Department of Plastic Surgery, The Second Affiliated Hospital of Soochow University, Suzhou 215006, China; ^2^Department of Plastic and Burn Surgery, The First Affiliated Hospital of Soochow University, Suzhou 215006, China

## Abstract

We report here the clinical, genetic, and molecular characteristics of type 2 diabetes in a Chinese family. There are differences in the severity and age of onset in diabetes among these families. By molecular analysis of the complete mitochondrial genome in this family, we identified the homoplasmic m.15897G>A mutation underwent sequence analysis of whole mitochondrial DNA genome, which localized at conventional position ten of tRNA^Thr^, and distinct sets of mtDNA polymorphisms belonging to haplogroup D4b1. This mutation has been implicated to be important for tRNA identity and stability. Using cybrid cell models, the decreased efficiency of mitochondrial tRNA^Thr^ levels caused by the m.15897G>A mutation results in respiratory deficiency, protein synthesis and assembly, mitochondrial ATP synthesis, and mitochondrial membrane potential. These mitochondrial dysfunctions caused an increase in the production of reactive oxygen species in the mutant cell lines. These data provide a direct evidence that a novel tRNA mutation was associated with T2DM. Thus, our findings provide a new insight into the understanding of pathophysiology of maternally inherited diabetes.

## 1. Introduction

Type 2 diabetes mellitus (T2DM) is a serious condition which is widespread in China, affecting upwards of 10% of the adult population [1, 2]. This disease is multifactorial in nature, arising in a heterogeneous fashion owing to interactions between both genetic and environmental factors [3]. As T2DM is a serious metabolic disease, and mitochondria serve as invaluable metabolic regulators within cells, these organelles are thought to be central to T2DM pathogenesis [4]. As multiple studies have found that T2DM risk can be transmitted in a matrilineal fashion, this further supports the potential for a mitochondrial role in the development of this serious disease [2, 5]. Indeed, some studies have proposed that mutations within mitochondrial tRNA sequences are key factors influencing the development of T2DM and other metabolic diseases [6]. The 3243A>G, m.3264T>C mutations in the tRNA^Leu(UUR)^, m.4291T>C mutation in the tRNA^Ile^, m.14709T>C, m.14692A>G mutations in the tRNA^Glu^, and m.10003T>C mutation in the tRNA^Gly^ have all been identified as potential mutations affecting T2Dm disease predisposition [7–12]. Exactly how these mutations ultimately contribute to disease onset, however, remains poorly understood, and as such, there is a clear need for additional research into how mitochondrial dysfunction can act to mediate the onset or progression of T2DM.

During a T2DM genetic screening effort among 100 Chinese subjects, we were able to detect a G-to-A transition at position 15897 in individuals with a familial history of matrilineal T2DM transmission, as well as a set of variants belonging to the Asian haplogroup D4b1. This mutation is likely to disrupt normal base pairing (10G-25C) for the tRNA^Thr^ DHU-stem, thereby resulting in its abnormal functionality and structure, thus likely resulting in mitochondrial dysfunction. Therefore, we should be to explore the function of this m.15897G>A mutation using cybrid cell lines in order to observe the effects of this mutation on mitochondrial functionality, with a particular focus on processes such as mitochondrial tRNA^Thr^ levels, protein synthesis and assembly, membrane potential, adenosine triphosphate (ATP) production, and reactive oxygen species (ROS) generation.

## 2. Materials and Methods

### 2.1. Subjects

We initially recruited a Chinese T2DM proband who was not related at the First Affiliated Hospital of Soochow University, China. After obtaining informed consent, we then conducted clinical evaluations and isolated blood samples from all family members who agreed to participate in this study. Any family or personal history of metabolic disease was identified through thorough historical and physical exams, while proband and family members also underwent blood glucose testing at our hospital. In total, we recruited 104 control Chinese volunteers in order to screen for any mitochondrial tRNA mutations identical to those identified in the family of T2DM patients. The Ethics Committees of Soochow University approved all study protocols. The principles of the Declaration of Helsinki were observed in this study [13].

### 2.2. Mitochondrial Genome Mutation Analysis

We isolated total genomic and mitochondrial DNA from patient whole blood samples via a DNA Extraction Kit (QIAGEN: 51104). We then conducted PCR amplification of the whole mitochondrial genome of the proband subject with T2DM (SZDM012-III-14), generating 24 overlapping PCR product fragments through the use of primers specific to the light and heavy strands, as in the previous studies [12]. Subsequently, we sequenced the fragments by an ABI 3700 automated DNA instrument and analyzed by comparing to the updated Cambridge consensus sequence (GenBank accession number: NC_012920) [14].

In order to detect the m.15897G>A mutation of the mitochondrial tRNA^Thr^ gene, we used the following primers in order to specifically amplify the 15792-31 region of the mitochondrial DNA via PCR: F:5′-TCA TTG GAC AAG TAG CAT CC-3′ and R:5′-GAG TGG TTA ATA GGG TGA TAG-3′, as in the previous reports [11]. We then isolated and assessed these fragments as in the previous reports [12].

### 2.3. Phylogenetic Analyses

In order to perform interspecific analyses, as in the previous studies, we compared human mtDNA nucleotide variation frequencies to sequences in 16 other species of vertebrates in order to determine the appropriate conservation index (CI) [15, 16]. Based on this CI value, it was then possible to determine what percentage of these 16 species exhibit a conserved wild-type base at the position of interest.

We further used an online application (http://www.mitotool.org/genomeRSRS.html) in order to determine the mitochondrial haplogroup of the proband and related individuals, consistent with current haplogroup nomenclature [17, 18].

### 2.4. Cell Culture

The Epstein-Barr virus was used to generate immortalized patient cell lines from the proband patient (III-3) bearing the m.15897G>A mutation, as well as from a control individual (C2). These cells were cultured in RPMI 1640 containing 10% FBS. Cybrid cells were generated by adapting previous protocols. Briefly, bromodeoxyuridine- (BrdU-) resistant 143B.TK-cells were cultured in DMEM containing 5% FBS, and the *ρ*^o^206 cell line lacking mtDNA derived from these same cells was also grown under these conditions in the presence of 50 *μ*g uridine/ml. The patient and control cell lines were then enucleated and fused with the *ρ*^o^206 cells. The resultant cybrid cell lines were then selected in uridine-free DMEM supplemented with BrdU, allowing for donor-derived cybrid lines that could then be assessed for the m.15897G>A mutation, amounts of mtDNA, and other cellular genetic features. The resultant cybrid lines were maintained in DMEM containing 5% FBS.

### 2.5. Mitochondrial tRNA Northern Blotting

For the tRNA Northern blot analysis, total mitochondrial RNAs were isolated as in previous reports [19, 20]. We then electrophoretically separated 2 *μ*g of this mitochondrial RNA onto a nylon membrane (Roche), prior to conducting a hybridization analysis using appropriate oligodeoxynucleotides as probes. We used digoxigenin- (DIG-) labeled probes specific to 5S RNA, tRNA^Ala^, tRNA^Ile^, tRNA^Thr^, tRNA^Gln^, and tRNA^His^, and then used these for hybridization reactions and subsequent densitometric quantification, as in the previous reports [19, 20]. For the tRNA mobility shift assay, 2 *μ*g of total mitochondrial RNAs was electrophoresed as detailed elsewhere. After electrophoresis, the gels were treated according to the procedure for the tRNA Northern blot analysis described above [19, 20]. Moreover, the aminoacylation of tRNA assays was performed as detailed previously [19, 20].

### 2.6. Western Blotting

Western blotting was conducted as in the previous studies [19, 20]. Primary antibodies used herein include those targeting ND6 (Abcam, ab81212), ND4 (Abcam, ab219822), ATP6 (Proteintech, 55313-1-AP), ND1 (Abcam, ab74257), CYB (Proteintech, 55090-1AP), CO2 (Abcam, ab110258), and VDAC (Proteintech, 10866-1-AP). As a secondary detection antibody, we employed the peroxidase AffiniPure goat anti-mouse IgG and goat anti-rabbit IgG (Jackson) constructs, and an enhanced chemiluminescence (ECL) system (Millipore) was used for band detection, after which densitometric calculations were performed.

### 2.7. Blue Native Electrophoresis Analysis

Blue Native gel electrophoresis (PAGE) was performed by isolating mitochondrial proteins from mutant and control cybrid cell lines as described elsewhere [21]. Samples containing 30 *μ*g of proteins were separated on 3~12% Native PAGE gel. The primary antibodies applied for this experiment were NDUFA9 (Abcam, ab128744), SDHA (Abcam, ab14715), UQCRC2 (Abcam, ab203832), COX5A (Abcam, ab110262), and ATP5C (Abcam, ab119686). As a secondary detection antibody, an ECL system was used for band detection, after which densitometric calculations were performed as shown in Western blotting.

### 2.8. Measurements of Enzymatic Activity

The complex I, II, III, and IV activities were assessed as described elsewhere [20].

### 2.9. Assessment of ATP Levels

We utilized a CellTiter-Glo Luminescent cell viability assay (Promega, G7572) in order to assess levels of mitochondrial ATP based on a modified protocol provided by the manufacturer, with additional previously detailed modifications [20].

### 2.10. Mitochondrial Membrane Potential Measurement

A JC-10 Mitochondrial Membrane Potential Microplate Assay Kit (Abcam, ab112134) was utilized based on the provided protocols, as in the previous studies, as a means of assessing the mitochondrial membrane potential [20]. Excitation/emission ratios of Ex/Em = 490/590 and 490/525 nm were determined as a means of measuring ΔΨm in samples, with the relative ratio of these two values in mutants and controls being calculated to determine the ΔΨm value.

### 2.11. ROS Measurements

The MitoSOX Red Mitochondrial Superoxide Indicator (Invitrogen, M36008) was used to assess ROS levels within live cells based on provided protocols, as in the previous studies [22]. ROS production rates were determined based on geometric mean intensities in samples, and ratios of these intensity values between unstimulated and H_2_O_2_-stimulated cells were used to determine whether ROS generation changed under oxidative stress conditions.

### 2.12. Statistical Analysis

Microsoft Excel 2016 was used for all statistical testing, comparing values via unpaired, two-tailed *t*-tests. *P* < 0.05 was the threshold of significance unless otherwise indicated.

## 3. Results

### 3.1. Familial T2DM Presentation in a Han Chinese Family

**Figure 1:**
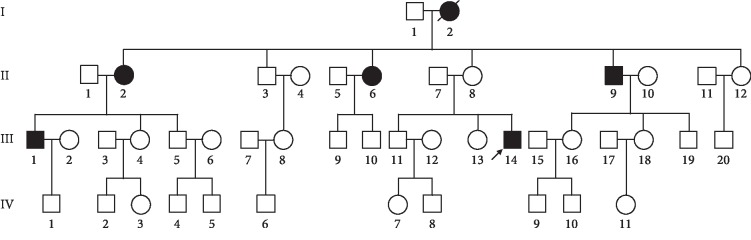
The Chinese pedigree with T2DM. The affected individuals are indicated by blackened symbols.

We identified a Chinese family SZDM012 that had visited the Diabetes Clinic at the First Affiliated Hospital of Soochow University (as shown in Figure 1). This patient underwent full physical examinations in an effort to identify any clinical or genetic factors associated with this instance of familial T2DM. All patients had been diagnosed with T2DM based on standard diagnostic criteria: (1) fasting plasma glucose ≥ 7 mmol/dL, (2) oral glucose tolerance ≥ 1.11 mmol/dL (200 mg/dL), or (3) ≥6.5% glycated hemoglobin (HbA1c). Patients were diagnosed as having an elevated diabetes risk if they did not have a history of diabetes and met the following criteria: (1) fasting plasma glucose from 5.5-7 mmol/L, (2) 2-hour plasma glucose levels from 0.78-1.11 mmol/dL (140-199 mg/dL), or (3) a HbA1c concentration of 5.7-6.4%. When a total of 10 matrilineal relatives were surveyed, a total of 6 were determined to have T2DM (3 males and 3 females; as shown in Table 1). T2DM symptoms were first diagnosed in these patients at the average age of 57 (range: 43-72), and other matrilineal relatives exhibited an elevated risk of diabetes based on the diagnostic criteria. We did not observe any other significantly abnormal clinical conditions in these related individuals, such as cardiac, muscular, or neurological diseases. No other surveyed members of this family exhibited T2DM, leading to our determination that this family exhibited matrilineally inherited T2DM.

### 3.2. Identification of T2DM-Associated Mitochondrial Mutations

**Figure 2:**
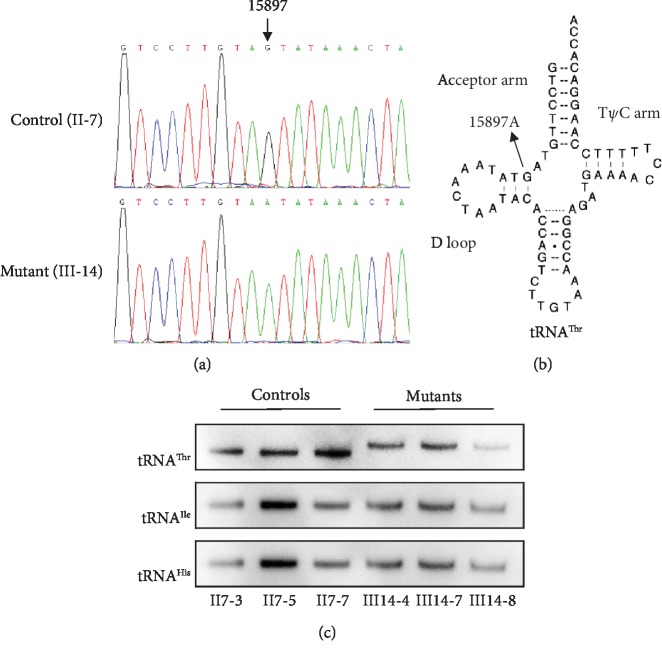
Identification of the m.15897G>A mutation in the tRNA gene. (a) Partial sequence chromatograms of tRNA gene from the proband and a Chinese control. An arrow indicates the location of the base changes at position 15897. (b) The location of the m.15897G>A mutation in the mitochondrial tRNA^Thr^. Arrow indicates the position of the m.15897G>A mutation. (c) Northern blot analysis of tRNAs under native conditions. Two *μ*g of total mitochondrial RNA from various cell lines was electrophoresed through native polyacrylamide gel, electroblotted and hybridized with DIG-labeled oligonucleotide probes specific for the tRNA^Thr^, tRNA^Ile^, and tRNA^His^, respectively.

We next sequenced the mitochondrial genomes of the proband SZDM012-III-14 patient in an effort to determine whether there were specific mitochondrial mutations underlying the observed matrilineal T2DM pattern of inheritance. After comparing our sequences from this patient to the Cambridge consensus sequence, as shown in Table 2, we detected a total of 46 single nucleotide polymorphisms in the mitochondrial genome and additionally determined their mitochondrial haplogroup to be the Eastern Asian haplogroup D4b1. Of these 46 mutations, 13 were previously described D-loop variants, 2 were known 12S rRNA gene variants, 2 were known 16SrRNA variants, one was the newly characterized m.15897G>A mutation in the tRNA^Thr^ gene (as shown in Figure 2), 27 variants (5 novel and 22 known) including 19 were to be silent variants, and 8 were missense mutations affecting protein-coding genes which were as follows: m.5178C>A(Leu>Met) in the *ND2* gene, m.7270T>C(Val-Ala) in the CO1 gene, m.8701A>G(Thr>Ala) and m.8860A>G(Thr>Ala) in the *ATP6* gene, m.10398A>G(Thr>Ala) in the ND3 gene, m.14256T>C(Ile>Val) in the *ND6* gene, and m.14766C>T(Thr>Ile) in the *CYTB* gene. These variants were evaluated for the pathogenicity using the following criteria: (1) present in <1% of the controls, (2) evolutional conservation, and (3) potential structural alterations. We next performed phylogenetic comparisons of these variants to known sequences of these same genes in 16 additional primate species in order to establish to what extent they were conserved. The known missense variants were reported polymorphic variation sites [6]. The two novel variants, CO1 m.7270T>C and ND6 m.14256T>C, revealed lowly evolutionary conservation. Therefore, these two variants were likely to be polymorphic. However, the m.15897G>A mutation in the tRNA^Thr^ gene exhibited highly evolutionary conservation. We also did not detect this m.15897G>A mutation in 100 Chinese control subjects, and we thus found no evidence that any detected mutation other than m.15897G>A was evolutionarily conserved.

### 3.3. Altered Conformation and Stability Level of tRNA^Thr^

We tested the conformation of tRNA^Thr^*in vivo* by native polyacrylamide gel. As shown in Figure 2(c), electrophoretic patterns demonstrated that the tRNA^Thr^ in three mutant cybrid cell lines carrying the m.15897G>A mutation migrated much slower than those of the control cybrid cell lines. In contrast, the levels of the tRNA^Ile^ and tRNA^His^ were similar with the control cybrid cell lines.

**Figure 3:**
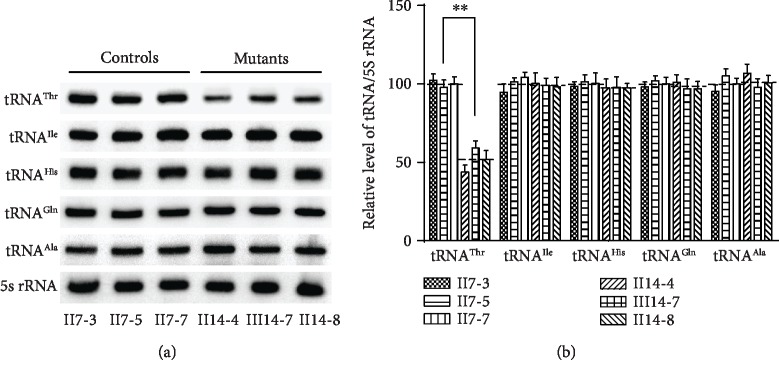
Northern blot analysis of mitochondrial tRNA. (a) Equal amounts of total mitochondrial RNA from various cybrid cell lines were electrophoresed through a denaturing polyacrylamide gel, electroblotted and hybridized with DIG-labeled oligonucleotide probes specific for the tRNA^Ala^, tRNA^Ile^, tRNA^His^, tRNA^Gln^, and tRNA^Ala^, respectively. (b) Quantification of tRNA levels. Average relative tRNA content per cell was normalized to the average content per cell of 5S rRNA in three mutant cybrid cell lines carrying the m.15897G>A variant and three control cybrid cell lines. The values for the latter are expressed as percentages of the average values for the control cell lines.^∗∗^*P* < 0.01 indicates the significance, according to the *t*-test, of the differences between mutant and control cell lines.

Furthermore, we found that mutant cell lines exhibited significantly decreased tRNA^Thr^ levels as compared with controls, with the 5S RNA used for normalization within cells (as shown in Figure 3). Indeed, mutant cells exhibited tRNA^Thr^ levels which were 51.53% (*P* < 0.05) of those in control cells following normalization. For comparison, these mutant cells had steady-state tRNA^Ile^, tRNA^His^, tRNA^Gln^, and tRNA^Ala^ levels of 99.19%, 97.45%, 98.30%, and 101.78%, respectively, relative to control cybrid cell lines (as shown in Figure 3).

### 3.4. Perturbed Aminoacylation of tRNA^Thr^

**Figure 4:**
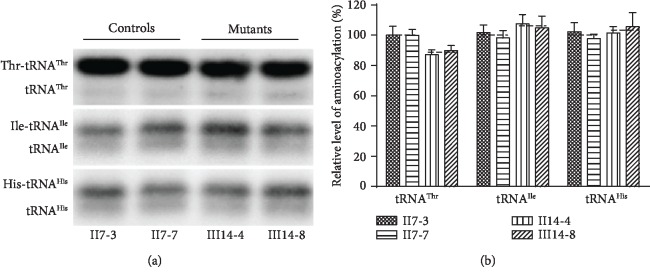
Aminoacylation assays. (a) Measurements of the aminoacylation levels *in vivo*. Two *μ*g of total mitochondrial RNA purified from various cell lines under acid conditions was electrophoresed, electroblotted, and hybridized with a DIG-labeled oligonucleotide probe specific for the tRNA^Thr^. The blots were then stripped and rehybridized with probes specific for the tRNA^Ile^ and tRNA^His^, respectively. Average relative tRNA^Thr^ aminoacylation (Thr-tRNA^Thr^) content per cell was normalized to the average content per cell of total tRNA^Thr^ (Thr-tRNA^Thr^+tRNA^Thr^) in mutant and control cybrid cell lines. (b) The proportion of aminoacylated tRNA^Thr^ in the mutant and control cybrid cell lines. The calculations were based on three independent determinations.

We investigated the aminoacylation properties of carrying m.15897G>A mutation cybrid cell lines. As shown in Figure 4, the efficiencies of aminoacylated tRNA^Thr^ in the mutant cell lines reflected 11.42% decrease, relative to the average values of control cell lines. However, the levels of aminoacylation in tRNA^Ile^ and tRNA^His^ in mutant cell lines were comparable with those in control cell lines.

### 3.5. Reduced Mitochondrial Protein Levels

**Figure 5:**
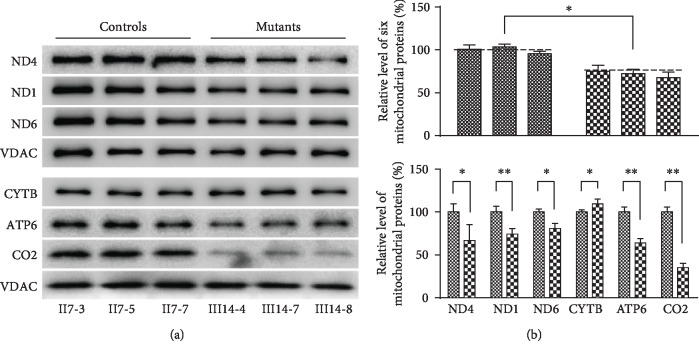
Western blot analysis of mitochondrial proteins. (a) 20 *μ*g of total cellular proteins from various cybrid cell lines was electrophoresed through a denaturing polyacrylamide gel, electroblotted and hybridized with respiratory complex subunits in mutant and control cells with VDAC as a loading control. (b) Quantification of 6 respiratory complex subunits. The levels of ND6, ND4, ATP6, CO2, and ND1 in two mutant cell lines and two control cybrid cell lines were determined. The error bars indicate two standard errors of the means. ^∗^*P* < 0.05 and ^∗∗^*P* < 0.01 indicate the significance, according to the *t*-test, of the differences between mutant and control cybrid cell lines.

We next assessed the levels of respiratory complex subunits encoded by the mitochondrial DNA via Western blotting, using VDAC to control for equal protein loading. As shown in Figure 5, we found that levels of four proteins generated by mitochondrial translation in mutant cells were present at average levels 71.71% relative to control cybrid cell lines (*P* < 0.05). Cells bearing the m.15897G>A mutation also exhibited significant 66.78%, 74.30%, 80.74%, 63.78%, and 35.31% reductions in levels of the proteins ND4, ND1, ND6, ATP6, and CO2, respectively. In contrast, the levels of the CYB protein were not significant with the control cybrid cell lines. However, the levels of polypeptide synthesis in mutant cells showed no significant correlation relative to those in control cells with either the number of codons or the proportion of threonine residues.

### 3.6. Imbalances in OXPHOS Complexes

**Figure 6:**
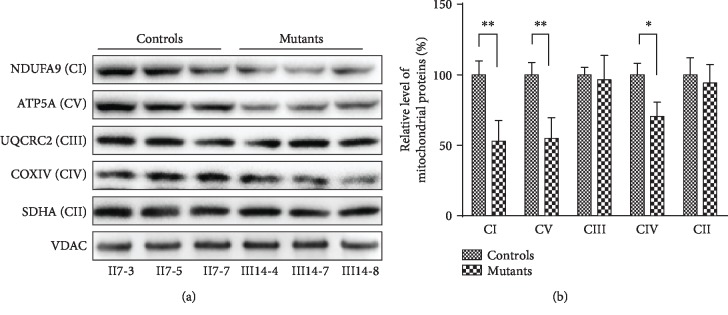
Imbalances in OXPHOS complexes. (a) The steady-state levels of five OXPHOS complexes by Blue Native gel electrophoresis. Fifteen *μ*g of mitochondrial proteins from mutant and control cell lines was electrophoresed through a Blue Native gel, electroblotted and hybridized with antibodies for NDUFA9, SDHA, UQCRC2, COX5A, ATP5C (complexes I, II, III, IV, and V), and VDAC as a loading control. (b) Quantification of levels of complexes I, II, III, IV, and V in two mutant cell lines and two control cybrid cell lines was determined. The error bars indicate two standard errors of the means. ^∗^*P* < 0.05 and ^∗∗^*P* < 0.01 indicate the significance, according to the *t*-test, of the differences between mutant and control cybrid cell lines.

We measured the steady-state levels of five OXPHOS complexes by Blue Native gel electrophoresis. As shown in Figure 6, the levels of complex I, complex IV, and complex V in the m.15897G>A mutation cell lines were 52.85%, 70.39%, and 54.93% of the levels observed in control cybrid cell lines, respectively. However, the levels of complexes II and III in the mutant cell lines were comparable with those of three control cell lines.

### 3.7. Mutation Led to Decreased Complex I and IV Activity

**Figure 7:**
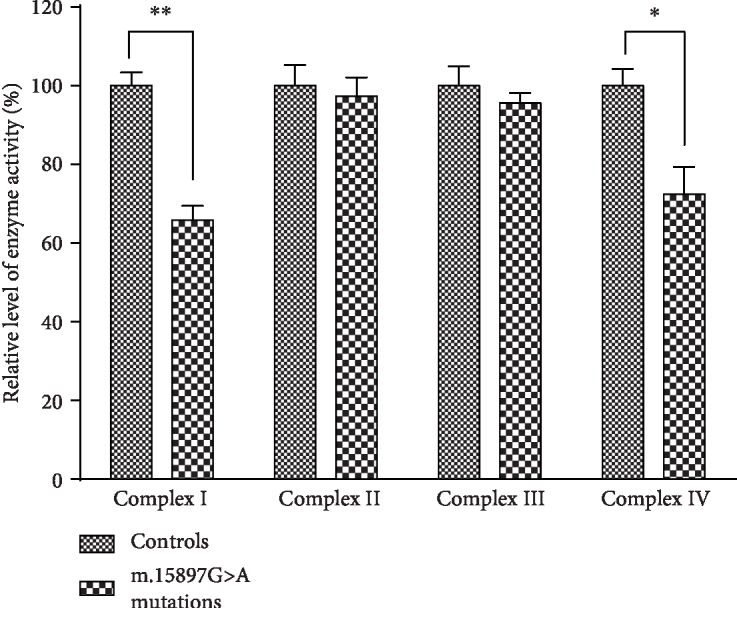
Respiratory complex activities. The activities of respiratory complexes were investigated by enzymatic assay on complexes I, II, III, and IV in isolated mitochondria from lymphoblastoid cell lines derived from the mutant and control cybrid cell lines. Activities of complexes I, II, III, and IV were normalized by citrate synthase activity. ^∗^*P* < 0.05 and ^∗∗^*P* < 0.01 indicate the significance, according to the *t*-test, of the differences between mutant and control cybrid cell lines.

We further assessed the consequences of this m.15897G>A mutation on oxidative phosphorylation using isolated mitochondrial from our mutant and control cybrid cell lines. As shown in Figure 7, we found that complex I and IV activity in the m.15897G>A mutant mitochondria was 70.63%, and 85.68% of the activity observed in control cybrid cell lines (*P* < 0.05), whereas no changes in complex II/III activity were observed.

### 3.8. ATP Generation Is Reduced in Mutant Cells

**Figure 8:**
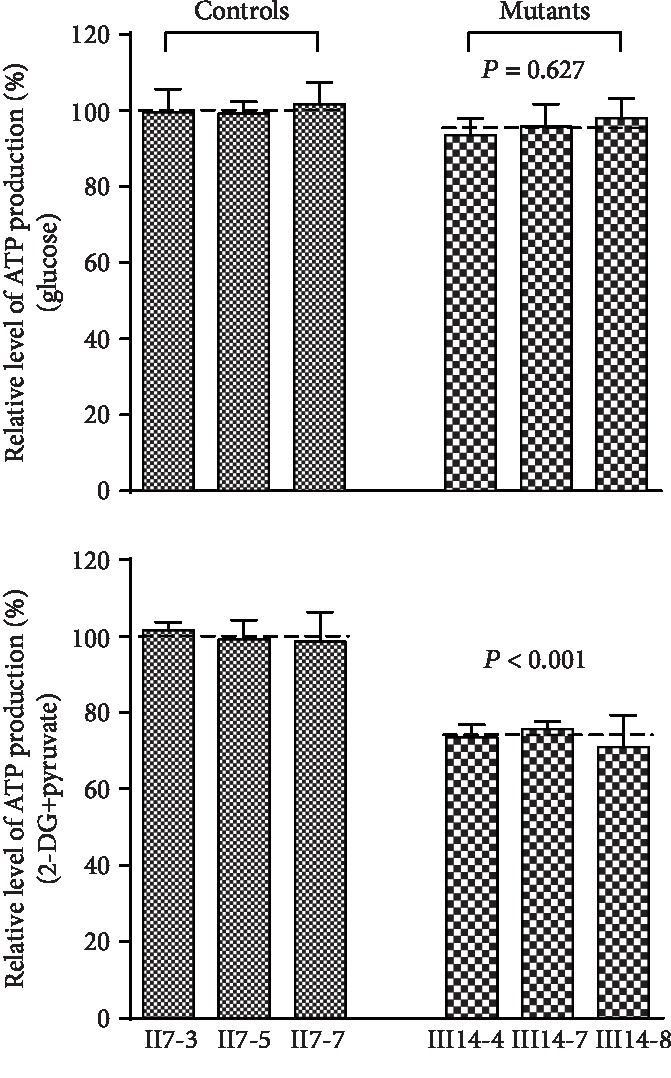
Mitochondrial ATP levels. Mutant and control cybrid cell lines were incubated with 10 mM glucose or 5 mM 2-deoxy-d-glucose plus 5 mM pyruvate to determine ATP generation under mitochondrial ATP synthesis. Average rates of ATP level per cell line in mitochondria are shown. The determinations were made for each cybrid cell line. The calculations were based on the independent determinations in each cell line. *P* indicates the significance, according to the *t*-test, of the differences between mutant and control cell lines.

We next assessed how this m.15897G>A influences ATP generation in the mitochondria of affected cells via a luciferin/luciferase assay system. Cells were combined with media containing glucose, with or without oligomycin (to inhibit ATP synthase and promote glycolysis) or a combination of pyruvate and 2-deoxy-d-glucose (to inhibit glycolysis and promote oxidative phosphorylation). As shown in Figure 8, we found that glycolysis activity was comparable between mutant and control cells, whereas ATP production via oxidative phosphorylation was significantly reduced in mutant cybrid cell lines relative to control cybrid cell lines, with ATP production on 70.99% and 75.68% (average 73.37%) that observed in control cells (*P* = 0.009).

### 3.9. Mitochondrial Membrane Potential Changes

**Figure 9:**
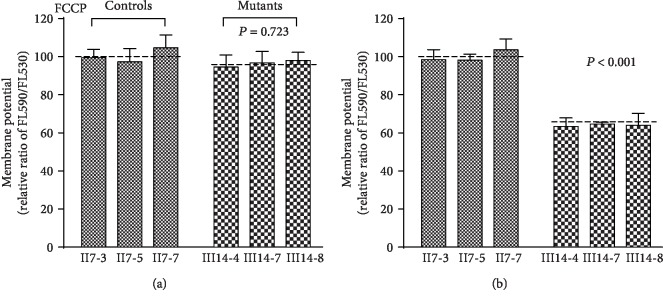
Mitochondrial membrane potential analysis. The mitochondrial membrane potential (ΔΨm) was measured in mutant and control cybrid cell lines using a fluorescence probe JC-10 assay system. The ratios of fluorescence intensities Ex/Em = 490/590 nm and 490/530 nm (FL_590_/FL_530_) were recorded to delineate the ΔΨm level of each sample. The relative ratios of FL_590_/FL_530_ geometric mean between mutant and control cybrid cell lines were calculated to reflect the level of ΔΨm. Relative ratio of JC-10 fluorescence intensities at Ex/Em = 490/530 nm and 490/590 nm in the absence and presence of 10 *μ*M of carbonyl cyanide 3-chlorophenylhydrazone (FCCP). The average of 3 determinations for each cybrid cell line is shown. *P* indicates the significance, according to the *t*-test, of the differences between mutant and control cell lines.

We next assessed how m.15897G>A mutations altered mitochondrial membrane potential (ΔΨm) with the help of the JC-10 fluorescent probe. As shown in Figure 9, we observed decreased ΔΨm values in mutant cells bearing the m.15897G>A mutation, with decreases averaging 64.04% of control (63.45% and 64.69%) (*P* < 0.05). We found that ΔΨm in mutant cells treated with FCCP were similar to values in control cells.

### 3.10. Elevated ROS Production

**Figure 10:**
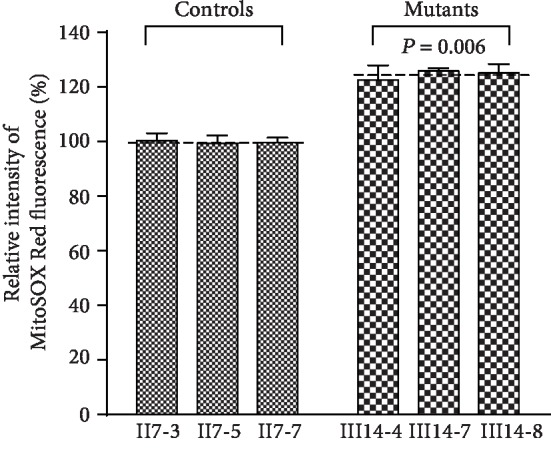
Ratio of geometric mean intensity. Measurement of mitoROS. The levels of ROS generation by mitochondria in living cells from mutant and control cybrid cell lines were determined using the mitochondrial superoxide indicator MitoSOX Red. The average of the determinations for each cybrid cell line is shown. The error bars indicate two standard errors of the means. *P* indicates the significance, according to the *t*-test, of the differences between mutant and control cell lines.

We used flow cytometry to assess ROS production by mutant and control cells but upon H_2_O_2_ stimulation and at baseline. As shown in Figure 10, we found that mutant cells bearing the m.15897G>A mutation exhibited increased ROS production, with an average increase of 124.37% (122.45% and 125.67%) relative to control (*P* < 0.05).

## 4. Discussion

Herein, we determined that the tRNA^Thr^ m.15897G>A mutation is associated with the matrilineal transmission of T2DM risk in a Chinese family. This m.15897G>A mutation affects coding at position 10 of this tRNA, and the presence of a guanine at this position is highly evolutionarily conserved among species, suggesting it is essential for normal tRNA function or pre-tRNA processing [23, 24]. We were only able to detect this mutation in members of a Chinese family exhibiting T2DM, and not in 100 control patients. As shown in Table 1, we observed impaired glucose tolerance in 6 (3 males/3 females) out of a total of 20 matrilineal relatives, compared with the average penetrances of T2DM in other Chinese families carrying the m.10003T>C and m.14692A>G mutation [11, 12]. Average age of diabetes onset in those with the m.15897G>A mutation was 57 (range: 43–72), while the average ages in those families with m.10003T>C and m.14692A>G mutations were 45 and 60 years, respectively [11, 12]. As families with identical m.15897G>A mutations exhibit significant variability, this suggests that nuclear genes and environmental factors (such as diet, BMI, and physical activity) also play a role in modifying phenotypic manifestations of T2DM in these individuals.

The observed mutation located in the 15897 position at a highly conserved nucleotide (conventional position 10) of tRNA^Thr^, where the position is important for the stability and identity of tRNA. The anticipated destabilization of base pairing (10G-25C) by the m.15897G>A mutation may affect secondary structure and function of this tRNA, as in the cases of tm.10003T>C and m.14692A>G mutations [11, 12]. We were able to utilize cell lines bearing this m.15897G>A mutation to provide clear evidence of multiple forms of mitochondrial dysfunction in these cells. For one, we found tRNA^Thr^ levels to be clearly decreased in these mutant cells relative to controls (51.53%). Furthermore, the altered tertiary structure caused by the m.15897G>A mutation may perturb the aminoacylation of tRNA^Thr^ by either charging inefficiently or mischarging with mitochondrial threonyl-tRNA synthetase. It is possible that this mutated form of tRNA^Thr^ has less metabolic stability, resulting in its more rapid degradation and thus reducing effective levels of the tRNA, as in the case of the of tRNA^Leu(UUR)^ m.3243A>G mutation in [25, 26]. This mutation was linked to significant reductions in 7 mitochondrially encoded proteins, leading to incomplete fully assembled OXPHOS complexes, and all of these reductions were associated with a marked reduction in ATP synthesis, as well as increased oxidative stress that may be linked to defects in normal energetic processes. The observed 26.63% decrease in mitochondrial ATP by cells bearing the m.15897G>A mutation was similar to that in cells with the m.14692A>G mutation [11]. Impaired respiratory chain activity can also result in impaired mitochondrial membrane potential, which is closely linked to cell viability, and we found clear reductions in this potential by roughly 35.96% in mutant cells. Moreover, we observed a ~28.29% reduction in mitochondrial protein levels in cells bearing this mutation, and these cells also exhibited altered complex I/IV enzymatic activity which may coincide with increased electron leakage and elevated ROS production. These defective mitochondrial activities were associated with excessive ROS generation in mutant cells, with a roughly 24.37% increase relative to controls. ROS production can in turn result in substantial damage to proximal proteins and other macromolecules, thereby impairing cell functionality.

In conclusion, our work is the first to detect a link between the m.15897G>A mutation and T2DM. Disrupted normal metabolism for this tRNA as a result of this mutation appears to significantly impair normal mitochondrial functionality, with impaired mitochondrial protein synthesis, mitochondrial complex enzymatic activity, membrane potential, ATP production, and ROS generation. As such, this m.15897G>A mutation is clearly a relevant candidate biomarker for heritable T2DM risk, making it useful for molecular diagnostics. Our results therefore have the potential to improve current understanding of T2DM, offering new insights into disease development and suggesting potential avenues for treatment (such as antioxidation therapy) or prevention.

## Figures and Tables

**Table 1:** Summary of clinical and biochemical data of members in a Chinese pedigree with T2DM and control subject (II-7).

Subjects	Gender	Age at onset (yrs)	Fasting glucose (mmol/L)	Oral glucose tolerance (mmol/dL or mg/dL)	HbA1c (%)
I-2	F	68	7.8	1.71 (295)	6.9
III-14	M	43	8.1	1.78 (320)	7.4
II-2	F	72	10.8	1.61 (280)	7.5
II-6	F	65	11.3	1.68 (285)	7.1
II-9	M	59	9.7	1.75 (315)	6.8
III-1	M	47	10.1	1.60 (280)	7.3
II-7	M	39	5.8	0.72 (129)	5.2

**Table 2:** mtDNA variants in a Chinese pedigree with T2DM.

Gene	Position	Replacement	AA change	Conservation (H/B/M/X)^a^	Previously reported^b^
D-loop	73	A-G			Yes
	263	A-G			Yes
	310	T-TC			Yes
	374	A-G			Yes
	489	T-C			Yes
	514	C-Del			Yes
	515	A-Del			Yes
	16185	C-T			Yes
	16189	T-C			Yes
	16223	C-T			Yes
	16232	C-A			Yes
	16319	G-A			Yes
	16362	T-C			Yes

12S rRNA	750	A-G		A/A/A/-	Yes
	1438	A-G		A/A/A/G	Yes

16S rRNA	2706	A-G		A/G/A/A	Yes
	3010	G-A		G/G/A/A	Yes

ND2	4769	A-G			Yes
	4883	C-T			Yes
	5178	C-A	Leu-Met	L/T/T/T	Yes

CO1	7028	C-T			Yes
	7270	T-C	Val-Ala	V/M/M/I	No

CO2	8020	G-A			Yes

ATP8	8414	C-T	Leu-Phe	L/F/M/W	Yes

ATP6	8701	A-G	Thr-Ala	T/S/L/Q	Yes
	8860	A-G	Thr-Ala	T/A/A/T	Yes

CO3	9540	T-C			Yes
	10136	A-G			No

ND3	10181	C-T			Yes
	10398	A-G	Thr-Ala	T/T/T/A	Yes
	10400	C-T			Yes

ND4	10807	A-G			No
	10873	T-C			Yes
	11242	C-T			Yes
	11719	G-A			Yes

ND5	12705	C-T			Yes

ND6	14256	T-C	Ile-Val	I/M/I/V	No
	14596	A-G			Yes
	14617	A-G			No
	14668	C-T			Yes

Cytb	14766	C-T	Thr-Ile	T/S/T/S	Yes
	14783	T-C			Yes
	15043	G-A			Yes
	15301	G-A			Yes

tRNA^Thr^	15897	G-A		G/G/G/G	No

^a^Conservation of amino acid for polypeptides or nucleotide for RNAs in human (H), bovine (B), mouse (M), and Xenopus laevis (X). ^b^CRS: Cambridge Reference Sequence. ^c^See online mitochondrial genome databases http://www.mitomap.org.

## Data Availability

The data used to support the findings of this study are available from the corresponding authors upon request.
